# Impact of government subsidies on innovation of Chinese biopharmaceutical firms: Based on kink threshold model

**DOI:** 10.3389/fpubh.2023.1087830

**Published:** 2023-02-23

**Authors:** Qing Li, Jie Di, Qingqing Liu

**Affiliations:** ^1^Center for Innovation Management Research of Xinjiang, Xinjiang University, Wulumuqi, China; ^2^School of Economics and Management, Xinjiang University, Wulumuqi, China

**Keywords:** government subsidies, biopharmaceutical firms, innovation, kink threshold, regional differences, enterprise ownership differences

## Abstract

Do government subsidies achieve the goals of stimulating firm innovation and macro-regulation? Existing studies have not reached a consistent conclusion. We will study the incentive effect of government subsidies on innovation of biopharmaceutical firms, analyze the optimal interval of government subsidies, and improve the efficiency of government subsidies. Thus, based on kink threshold model using data from Chinese biopharmaceutical listed companies from 2013–2019, this study analyzes the impact of government subsidies on innovation inputs and outputs. Government subsidies can stimulate innovation inputs and outputs of biopharmaceutical firms. Meanwhile, such subsidies have a significant threshold effect on innovation inputs and outputs, and there is an optimal interval effect. Additionally, concerning enterprise ownership, government subsidies have a more significant role in promoting innovation of non-state biopharmaceutical firms. Regarding regional differences, such subsidies have a more significant role in promoting innovation of firms in the less economically developed central and western regions. This study reveals the influence pattern of government subsidies, and provides insights and suggestions to formulate subsidy policies and enhance innovation.

## Highlights

- Biomedical industry is one of the important technology-intensive industries, it has played an important role in solving the health, environmental, and resource problems faced by human development and. Government subsidies play an important role in raising the firms' level of innovation and enhancing the sustainability of firms.
- Government subsidies have threshold effects on firms' innovation, and the study compares the difference between static and dynamic threshold models.
- There are enterprise ownership and regional differences in the in and effect of government subsidies.

## 1. Introduction

Innovation is the basis to lead companies to improve their market competitiveness and sustainability (1). The 21^st^ century is an era of technological innovation, leading transformation from a production-oriented to a technology-oriented economy (2). The biomedical industry is one of the important technology-intensive industries (3), involving biopharmaceutical, intelligent medical treatment and medical equipment, and other biomedical industries (4). It has played an important role in solving the health, environmental, and resource problems faced by human development (5, 6). In particular, the role in improving the general health of population is becoming increasingly significant. Currently, the demographic structure of China is characterized by the longevity and aging of the population (7). Facing chronic and serious diseases that may accompany the healthy development of human life, and other health problems that aging may bring, people's awareness of health care is increasing (8) and the demand for medical products and services is growing (9), driving the rapid development of the biopharmaceutical industry. Since the outbreak of COVID-19 in 2020, repeated outbreaks have posed a threat to global economic development and the safety of human life (10). Biomedical technology has become an important technological means to deal with health emergencies (11), and the biomedical industry is becoming one of global concern. In the context of a healthy China, the Chinese government has focused on increasing biotechnology research and development (R&D) and promoting the rapid development of biomedical firms with biotechnology as a pillar industry (12). In recent years, numerous research-based companies have emerged in the Chinese biomedical industry, consistently increasing investment in research personnel and funding (13) to protect their intellectual property rights and strengthen their R&D activities (14), thus advancing the generation of novel drugs, molecules, and patents (15). According to the statistical yearbook data, in 2019, the research and experimentation expenditure of industrial pharmaceutical manufacturing firms above the scale was about 60.96 billion yuan, which is 75.3% higher than in 2013, with about 122,720 R&D personnel and 32,296 development projects, with an annual growth rate of world sales of more than 30%. The Chinese biopharmaceutical industry is accelerating to become the next hardcore technology industry (16).

Government subsidies have been found to play a role in easing financial pressure and reducing R&D costs and risks for biopharmaceutical companies (17), thus stimulating their incentive to innovate. Moreover, government subsidies not only bring direct R&D funds to firms, but also lead to the recognition of the level of R&D (18, 19). Firms can send signals to the outside world to attract social investment, thus helping them lighten external financing constraints (20) and broaden their access to innovation resources. The relationship between government subsidies and enterprise innovation has been a hot topic in academic research, and research on the impact of government subsidies on innovation in high-tech industries can be summarized into four categories. First, most scholars believe that government subsidies have a promotional effect on enterprise innovation and can encourage firms to enhance their innovation motivation (21), expand the scale of R&D investment, and improve firms' innovation performance (22). Second, some argue that government subsidies have a crowding-out effect on enterprise innovation (23), and firms obtain government subsidies to squeeze out the original capital investment and fail to achieve the expected effect, thus showing a restraining effect (24). Third, some argue that there is a complex non-linear relationship between government subsidies and firm innovation. This non-linear relationship may be “U” or inverted “U” (25), or the threshold effect (26) is that more government subsidies are not better, but there is an optimal value (27), and subsidies will promote firm innovation when they reach the optimal value. Fourth, a few scholars argue that the effect of government subsidies on firm innovation is insignificant or ineffective (28, 29).

There is currently a proliferation of scholarly research on the biopharmaceutical field, with studies on the strategic performance of biopharmaceutical companies (30, 31), the development of technological innovation in the biopharmaceutical industry (32–34), and the impact of epidemics on biopharmaceutical companies (5). Fewer studies combine government subsidies on biopharmaceutical firm innovation (35, 36), fewer use the threshold models (37) to investigate the field, and even fewer focus on the sustainability of firm innovation using dynamic models (38). In recent years, various preferential policies of the government have played an important role in the development of biopharmaceutical firms. Studying the impact of government subsidies on biopharmaceutical firms' innovation can help provide valuable suggestions for government departments to better guide the development of firms, and can help put forward development suggestions for such firms to cope with new opportunities and challenges in the context of epidemics for reference. Therefore, based on existing studies, this study explores the impact of government subsidies on the innovative development of biopharmaceutical firms using panel data of biopharmaceutical firms in China from 2013–2019. The impact of government subsidies on the innovative development of biopharmaceutical firms is empirically tested through linear and static and dynamic threshold models. Additionally, the influence from the aspects of enterprise ownership and regional differences are analyzed.

## 2. Theory and hypotheses

### 2.1. Impact of government subsidies on innovation inputs

From the perspective of innovation inputs, government subsidies can directly help firms solve the problem of insufficient funds for innovation inputs (39). On the other hand, they can help firms alleviate the difficulties of financing constraints caused by market failure (40) and indirectly help firms obtain innovation resources. Carboni (41) and Mei (42) found that government subsidies can increase firms' external resources and greatly stimulate R & D investment. Kang and Park (43), Huergo and Moreno (44), and Wang et al. (45) established that government subsidies can make R&D investment more sufficient and encourage firms to expand their R&D investment scale. Government subsidies also play an important signaling role (46), which indirectly improves the information asymmetry between firms and external investors and helps firms obtain external financial assistance (47) or enter technological cooperation, thus improving firms' existing resources for innovation inputs. In contrast, Asker and Baccara (48) highlighted that firms would become overly dependent on government subsidies and less motivated to innovate. Jourdan and Kivleniece (49) found that firms' access to large-scale subsidies may change in the direction of increasing other benefits, creating a crowding-out effect. Recently, many scholars have proposed a non-linear relationship between government subsidies and firms' innovation investment. Wu et al. (50) revealed an inverted U-shaped relationship between the subsidy scale of new-energy firms and firms' innovation investment. Li et al. (38) demonstrated that government subsidies have a threshold effect on firms' innovation investment, and they only play a role in promoting innovation when they are in the right interval. Taken together, most scholars now believe that government subsidies can help firms relieve financial pressure and attract external investment, thus promoting innovation investment. As a result, the following hypothesis is proposed:

H1a: Government subsidies may significantly promote innovation investment of biopharmaceutical firms.
H1b: Government subsidies may have a significant threshold effect on biopharmaceutical firms' innovation investment.

### 2.2. Impact of government subsidies on innovation outputs

From the perspective of innovation output, government subsidies can increase output by providing firms with innovation resources and help them reduce R&D costs and risks, thus increasing their innovation initiatives (51). Li (52) highlighted that the R&D cycle of biopharmaceutical new products is usually 2–3 years or even longer, the R&D process requires extensive time and capital costs, and any capital chain breaks will bring risks to the firms, so biopharmaceutical firms need more support and protection from government policies. Bronzini and Piselli (53) established that the R&D subsidy program of the Italian northern regions has a significant impact on firms' innovation patent output. Plank and Doblinger (54) argued that direct financial support from public R&D funding is effective in increasing the number of patents in the renewable energy sector. Shinkle and Suchard (55) noted that government subsidies increase the external investment available to firms, which facilitates their innovation output. Hewitt-Dundas and Roper (56) argued that public support can increase firms' innovation output and innovation quality. However, Czarnitzki et al. (57) found no significant effect of government subsidies on R&D and patents. Xu et al. (58) also verified this view, and Wu et al. (59) suggested that government subsidies only fill the deficiency of firms' innovation funds and do not play a positive role in promoting firms' innovation output. Using a threshold model, Wei et al. (37) determined that government R&D subsidies have a double threshold effect on firms' innovation output, and government subsidies will play a positive role in promoting innovation output only when they are in the appropriate range. Synthesizing the above analysis, there may be various possible scenarios for the impact of government subsidies on innovation output of biopharmaceutical firms, and the following hypotheses are proposed:

H2a: Government subsidies may have a significant promoting effect on biopharmaceutical firms' innovation output.
H2b: Government subsidies may have a significant threshold effect on the innovation output of biopharmaceutical firms with significant interval incentive effects.

### 2.3. Heterogeneity of government subsidies and firm innovation

#### 2.3.1. Enterprise ownership level

Enterprises can be divided into state and non-state enterprises based on ownership, and these firms differ significantly in their internal governance models and access to government incentives and innovation strategies (60). Compared to non-state enterprises, state enterprises may have closer ties with government departments and easier access to policy information and valuable resources (61). Non-state enterprises have less direct access to subsidized resources, and the pressure of competition in the enterprise market leads to higher capital pressure and high costs (58), so the innovation risk has a greater impact on their business problems. Bai et al. (62) argued that government subsidies promote state enterprises more significantly. By contrast, Zhang (63) argued that there is a waste of resources within state enterprises, leading to inefficient effects of government subsidies on technological innovation. Xu et al. (58) revealed a positive relationship between government R&D subsidies and R&D investment in non-state biopharmaceutical firms only. Taken together, these findings lead to the following hypotheses:

H3a: The effects of government subsidies on biopharmaceutical firms' innovation at the ownership level may differ.
H3b: Government subsidies may have a significant threshold effect on the innovation inputs and outputs of state and non-state biopharmaceutical firms.

#### 2.3.2. Regional differences

The regional economic and innovation environment has a significant impact on the effect of government subsidies, such as human capital, technical support, and institutional protection (64). Therefore, there are regional differences in the intensity and effectiveness of government subsidies. In China, the eastern regions are relatively economically developed, more market-oriented, and have a better infrastructure (60). Firms in these regions are more internally developed and can take advantage of regional resources to improve the efficiency of firm innovation more easily (65). While the central and western regions are less developed economically, innovative technologies are often not sufficiently advanced, and regional advantages are not obvious (66). It is difficult for enterprises to seek development with regional industrial advantages. This leads to high financing constraints and capital pressure, and government subsidies are more likely to serve as a supplement to enterprises' innovation resources (67). Li et al. (38) argued that to optimize firms' resource allocation, the government implements preferential policies and financial support for the central and western regions, and even invests more funds. At present, the clustered distribution of China's biopharmaceutical industry has further emerged, and the unbalanced regional development has been further highlighted. An initial industrial spatial pattern of rapid development in the eastern regions has been formed, and the gap between the more economically developed eastern regions and the central and western regions continues to widen. Based on the above analysis, the following hypotheses were proposed:

H4a: There may be regional differences in the impact of government subsidies on biological firms' innovation.
H4b: Government subsidies may have a threshold effect on the innovation input and output of biomedical firms in the eastern and central and western regions.

**Figure 1:**
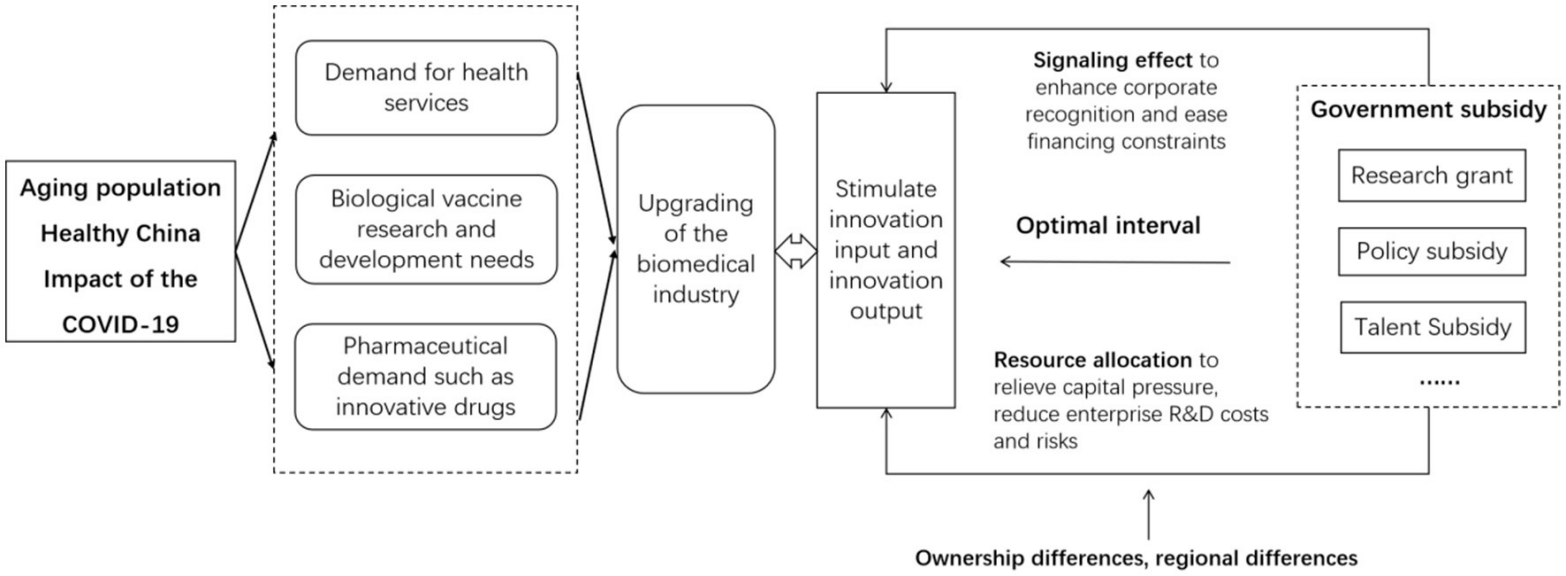
Theoretical model diagram.

Based on the above theoretical analysis and assumptions, we constructed the theoretical model diagram shown in Figure 1.

## 3. Methodology

### 3.1. Data

In this study, several financial databases such as Flush Finance and Sina Finance are integrated, and pharmaceutical manufacturing, medical device, biosafety, biological vaccine, smart medical, medical beauty, and other A-share concept companies in Shanghai and Shenzhen are selected as research samples. Corporate financial data come from the CSMAR database, and some missing data are supplemented by consulting the listed companies annual reports, where patent data come from the CNKI patent database. Considering the completeness and accuracy of the obtained enterprise data, this study refers to the research practices of Peng et al. (29) and Wu et al. (51), using STATA15.1 to preprocess the financial data and key variable data of listed companies. First, to truly reflect the situation of firms, the abnormal situation of firms such as losses and delisting during the period were excluded. Second, we deleted the samples with missing values of key variables for more than 2 years, and make up the rest by 0. Data from 251 biopharmaceutical companies from 2013–2019 were obtained after data processing, with a total of 1,757 valid observations. In addition, to avoid possible errors in the results due to the extreme values of variables, a tailing process for the control variables was created by Winsorizing before the empirical analysis, and the data located below the 1% and above 99% levels were replaced.

### 3.2. Models

#### 3.2.1. Basic regression model

Models (1) and (2) are applied to test the effects of government subsidies on innovation inputs and outputs. A fixed-effects model is used for R&D inputs. Because Patent is a count variable, it does not conform to the general assumption of a normal distribution in the linear model but is more adapted to the Poisson distribution in the generalized linear model. In addition, since the variance of patents in the sample is much larger than the expected value, negative binomial regression is chosen to estimate the firm's innovation output problem in this study.


(1) $$\begin{array}{l} \begin{array}{l} RDI_{it}=\alpha_{0}+\alpha_{1}Sub\_amount+\alpha_{2}\sum Con_{i,t}+{\text{λ}}_{i}+\eta_{t}\;\\ \;+\varepsilon_{i,t} \end{array} \end{array}$$

(2) $$\begin{array}{l} P{\text{atent}}_{it}=\beta_{0}+\beta_{1}Sub\_amount+\beta_{2}\sum Con_{i,t}+{\text{λ}}_{i}+\eta_{t}\;\\ \;+\varepsilon_{i,t} \end{array}$$

where *i, t* denote firm and year, represents firm innovation input and innovation output, respectively, *Sub*_*amount* is the total government subsidy received by the firm, *Con* represents the control variables, including *A*ge, *Size, Lev, Roe, Market, Holder, Fix, State*, λ, η denote individual and year fixed effects, and ε denotes the model random disturbance term.

#### 3.2.2. Threshold models

Based on theoretical analysis, a threshold model is developed to test whether there is a threshold effect of government subsidies on firms' innovation inputs and outputs. Existing studies mostly consider Hansen's (68, 69) static threshold models or test the non-linear relationship between variables by adding quadratic terms of explanatory variables. Seo and Shin (70) propose a dynamic panel threshold model based on differential generalized method of moments (GMM) estimation, which can not only solve the threshold problem well, but can also solve the endogeneity problem that exists in the regression volume.

Seo defines the dynamic panel threshold regression model as:


(3) $$\begin{array}{l} y_{it}=(1,{x^{\prime }}_{it})\varphi_{1}1\{q_{it}\leq \gamma \}+(1,{x^{\prime }}_{it})\varphi_{2}1\{q_{it}>\gamma \}+\delta_{it},i=1,\ldots \;N,\;\\ \;t=1\ldots ,T \end{array}$$

(4) $$\begin{array}{lll} \delta_{it} & = & {\text{λ}}_{i}+\varepsilon_{it} \end{array}$$

where *y*_*it*_ is the dependent variable, ${x^{{}^{\prime }}}_{it}$ is a *k*_1_ × 1 time-varying autoregressive vector that may contain a lag term *y*_*it*−1_, and 1{ · } is an indicator function. The value is determined from the relationship between the threshold variable *q*_*it*_ and the threshold γ; if true, the value is 1, otherwise 0. ϕ_1_ and ϕ_2_ are the slope parameters associated with different conditions. δ_*i*_ is a random perturbation term consisting of an individual fixed-effect λ_*i*_ and a zero-mean special random perturbation term ε_*it*_.

To deal with the presence of individual effects in the model, a first-order difference treatment is applied to Model 3:


(5) $$\begin{array}{l} \text{Δ}y_{it}=\alpha^{{}^{\prime }}\text{Δ}x_{it}+\beta^{{}^{\prime }}\text{Δ}X_{it}^{{}^{\prime }}1_{it}\left(\gamma \right)+\text{Δ}\delta_{it},\text{ Δ}\delta_{it}=\text{Δ}\varepsilon_{it} \end{array}$$

among them,


$$\begin{array}{l} \begin{array}{lll} \;\underset{k_{l}\times l}{\alpha } & = & {\left(\varphi_{1,2,\ldots ,}\varphi_{l,k_{l}+1}\right)}^{\prime },\underset{(k_{l}\times l)\times l}{\beta }=\varphi_{2}-\varphi_{1},\underset{2\times (l+k_{l})}{X_{it}}=\left[\begin{array}{c} \left(1,x_{it}^{\prime }\right)\\ \left(1,x_{it-1}^{\prime }\right) \end{array}\right],\\ \underset{2\times l}{1_{it}(\gamma )} & = & \left[\begin{array}{c} 1\{q_{it}>\gamma \}\\ -1\{q_{it-1}>\gamma \} \end{array}\right]. \end{array} \end{array}$$

Seo also derived an improvement for the possible discontinuity case of the threshold model, noting that a model with discontinuities can be made continuous when a value *k* exists such that (1, *x*)φ_2_ = *k*(*q*_*it*_ − γ) holds, which requires that *q*_*it*_ be an element in ${x^{{}^{\prime }}}_{it}$. At this point, Model 3 can be organized as follows:


(6) $$\begin{array}{l} \begin{array}{lll} y_{it} & = & \sigma_{1}+\sigma_{2}x_{it}^{{}^{\prime }}+k(q_{it}-\gamma )1\{q_{it}>\gamma \}+{\text{λ}}_{i}+\varepsilon_{it},\; \end{array}\;\\ \;\begin{array}{lll} i & = & 1,...N,t=1,...,\;T \end{array} \end{array}$$

Ultimately, the kink threshold model for this study is established as:


(7) $$\begin{array}{l} RDI_{it}=\sigma_{1}+\sigma_{2}x_{it}^{{}^{\prime }}+k\left(q_{it}-\gamma \right)1\left\{q_{it}>\gamma \right\}+{\text{λ}}_{i}+\varepsilon_{it} \end{array}$$

(8) $$\begin{array}{l} Patent_{it}=\sigma_{1}+\sigma_{2}x_{it}^{{}^{\prime }}+k\left(q_{it}-\gamma \right)1\left\{q_{it}>\gamma \right\}+{\text{λ}}_{i}+\varepsilon_{it} \end{array}$$

Taking *Sub*_*amount* as the threshold variable, ${x^{{}^{\prime }}}_{it}$ contains explanatory and control variables, and the number of iterations in the experiment is 400. When the model contains the lags *l*.*RDI, l*.*Patent* of the explained variables, Models 7 and 8 become models with dynamic effects. In the empirical analysis, a comparative analysis of the static and dynamic models is performed.

### 3.3. Variables

#### 3.3.1. Dependent variables

Innovation input (RDI) and innovation output (Patent) are selected as dependent variables. The former indicates the innovation process of firms and the latter the achievements of firms. Choosing innovation input and output together can measure firms' innovation levels more comprehensively. Existing literature uses the methods of total R&D investment, total R&D investment/operating income (71) and total R&D investment/total assets (58) to measure innovation input indicators. Therefore, this study uses the ratio of total R&D investment to total assets to express enterprise innovation investment RDI. The number of newly added patents not only reflects the activity and innovation ability of firms in the business process (72), but also has the advantages of unified patent identification standards and strict examination (73). Therefore, according to the practice of most scholars, this study uses the total number of patent applications to measure firms' innovation output (74, 75).

#### 3.3.2. Explanatory variables

The total amount of government subsidies (Sub_amount) is selected as the independent variable. This refers to the total amount of subsidies received by firms, including financial discounts, financial concession subsidies, research funding, and various special types. Although government subsidies are usually divided into multiple purposes and support firms through many specific projects, the authors argue that firms as a whole are linked from project to project and between each subsidy to a certain extent, and that each subsidy contributes to the innovation and development of firms through direct or indirect forms. Therefore, the total amount of government subsidies is selected to study its impact on innovation and development of pharmaceutical manufacturing firms, and the natural log is taken for the total amount of subsidies.

#### 3.3.3. Control variables

Firm characteristics and financial indicators such as firm age (Age), firm size (Size), solvency (Lev), profitability (Roe), market competition (Market), shareholding concentration (Holder), capital utilization (Fix), and enterprise ownership (State) were selected as control variables, and the variables are presented in Table 1. Firm age, ownership, and size are the most significant heterogeneous characteristics of firms (76). Firms accumulate experience along with their age to better develop innovation. Different enterprises, such as state and non-state enterprises, will have different concepts of innovation and innovation infrastructure, which will ultimately affect their innovation. Some studies demonstrate that compared to smaller firms, larger firms are better able to cope with various risk challenges and tend to perform better (77). In terms of financial governance, the corporate gearing ratio and return on net assets are selected to measure solvency and profitability, market competition, and shareholding concentration are selected to measure external and internal risk levels, respectively, and the fixed asset ratio is selected to measure capital utilization efficiency.

**Table 1:** Variable definitions.

**Variable type**	**Variable**	**Variable definition**
Dependent variables	RDI	Total R&D investment/Total assets
	Patent	Total number of patents
Explanatory variables	Sub_amount	Natural logarithm of total amount of government subsidies
Control variables	Age	Years since company has been listed
	Size	Natural logarithm of total assets
	Lev	Ratio of corporate total debt to total assets
	Roe	Ratio of net profit to net asset ^*^ 100%
	Market	Ratio of revenue to operating cost
	Holder	Ratio of the largest shareholder (%)
	Fix	Ratio of fixed assets to total assets (%)
	State	Non-state enterprises 0, state enterprises 1
	Area	More economically developed eastern regions 0, less economically developed central and western regions 1

### 3.4. Descriptive statistics and pairwise correlations of variables

The descriptive statistics of the variables are presented in Table 2. Among them, the maximum value of innovation input is 16.155 and the minimum value is 0.006, which indicates a large difference in the level of innovation input between firms. In comparison, the mean value of state enterprises (2.14) is slightly smaller than that of non-state enterprises (2.533). Patent applications range from 0–1,266, which indicates that there are significant differences in innovation output. In comparison, the mean value of state enterprises (61.784) is larger than that of non-state enterprises (38.529), indicating that the overall R&D output of state enterprises is greater; however, the difference between state enterprises (standard deviation 112.165) is even larger. The standard deviation of enterprise size (Size), enterprise solvency (Lev), and profitability (Roe) is less than 1, while that of the shareholding ratio of the largest shareholder (Holder) and fixed asset utilization rate (Fix) is 13.256 and 11.838, respectively. This indicates that there are large differences in the degree of fluctuations among the data, but they all conform to the law of normal distribution.

**Table 2:** Descriptive statistics of variables.

**Variable**	**All samples**				**State enterprises**		**Non-state enterprises**	
	**Mean**	**S. D**.	**Max**	**Min**	**Mean**	**S. D**.	**Mean**	**S. D**.
RDI	2.442	1.934	16.155	0.006	2.140	2.142	2.533	1.858
Patent	43.916	84.545	1,266	0	61.784	112.165	38.529	73.435
Sub_amount	16.533	1.349	20.401	9.752	16.763	1.663	16.463	1.230
Age	10.585	6.562	25.000	1.000	17.015	5.208	8.647	5.626
Size	22.100	0.985	24.850	20.231	22.554	0.996	21.963	0.940
Lev	0.353	0.178	0.800	0.046	0.424	0.178	0.332	0.173
Roe	0.077	0.091	0.287	−0.397	0.079	0.087	0.076	0.092
Market	2.104	1.209	7.728	1.080	1.726	0.844	2.218	1.278
Holder	31.832	13.256	68.530	7.770	33.998	14.403	31.179	12.824
Fix	19.287	11.838	52.920	1.152	19.340	12.022	19.271	11.787
State	0.232	0.422	1.000	0.000	1.000	0.000	0.000	0.000
Area	0.319	0.466	1.000	0.000	0.231	0.422	0.345	0.476
Observations	1,757	1,757	1,757	1,757	407	407	1,350	1,350

The correlation test between the variables (Appendix Table 1) reveals that the absolute values of the correlation coefficients among the explanatory variables were all less than 0.8. Moreover, the inflation factor value of each variable is below 10, and the tolerance is above 0.1. Therefore, multicollinearity among variables can be excluded.

## 4. Empirical analysis and results

### 4.1. Basic regression

The regression results of government subsidies on firms' innovation inputs and outputs are reported in Table 3. Columns (1) and (2) present the estimated results without the control variables, while columns (3) and (4) are those with the control variables. The results indicate that the regression coefficients of government subsidies on innovation inputs and outputs are significantly positive, and the coefficients of the core explanatory variables do not change significantly with the inclusion of the control variables. When the explanatory variables are innovation inputs and innovation outputs respectively, the coefficients of government subsidies are 0.118 and 0.228, indicating that for every 1% increase in government subsidies, innovation inputs will increase by 0.118% and innovation outputs will increase by 0.228%. The above results indicate that, government subsidies play the role of resource allocation. They can stimulate innovation in biopharmaceutical firms by increasing R&D funding, and achieving the goal of promoting firms' innovation inputs and innovation outputs. Therefore, hypotheses H1a and H2a were tested. About control variables, *Size, Lev, and Roe* are negatively related to *RDI*, while *Age, Market, and Fix* are positively related to *RDI*, indicating that in practice, variables may have both positive and negative moderating effects on the dependent variable.

**Table 3:** Basic regression.

**Variables**	**(1)**	**(2)**	**(3)**	**(4)**
	**RDI**	**Patent**	**RDI**	**Patent**
Sub_amount	0.059^**^	0.283^***^	0.118^***^	0.228^***^
	(0.028)	(0.016)	(0.028)	(0.022)
Age			0.265^**^	−0.033^***^
			(0.125)	(0.006)
Size			−0.675^***^	0.300^***^
			(0.138)	(0.041)
Lev			−0.015	0.056
			(0.320)	(0.196)
Roe			−0.008	0.420
			(0.416)	(0.311)
Market			0.191^***^	−0.138^***^
			(0.069)	(0.024)
Holder			0.007	0.008^***^
			(0.006)	(0.002)
Fix			0.007	−0.008^***^
			(0.005)	(0.002)
State			−0.320	0.363^***^
			(0.237)	(0.082)
Constant	1.419^***^	−1.288^***^	12.314^***^	−6.666^***^
	(0.460)	(0.260)	(3.272)	(0.735)
Observations	1,757	1,757	1,757	1,757

^***^, ^**^, and ^*^denote *p* < 0.01, *p* < 0.05, and *p* < 0.1, respectively. Standard errors are in parentheses.

### 4.2. Static and dynamic threshold regression results

According to Table 4, first, government subsidies as a threshold variable passed the threshold examination at the 1% significance level, indicating that there is a significant threshold effect of government subsidies on both innovation inputs and outputs of biopharmaceutical firms. Therefore, hypotheses H1b and H2b were tested. Secondly, as shown in the static model, when the Sub_amount < 17.591, government subsidies are negatively correlated with innovation input, indicating that for every 1% increase in government subsidies, innovation investment will decrease by 0.015%. When the Sub_amount > 17.591, it significantly promotes the firms' innovation input at the 10% level with a correlation coefficient of 0.497, indicating that for every 1% increase in government subsidies, innovation investment increases by 0.497%. It shows that the higher the government subsidy, the more it helps to stimulate enterprises' innovation investment; while too low government subsidies will easily trigger a crowding-out effect. In terms of innovation output, the threshold value is 15.867, when the Sub_amount < 15.867, government subsidies are negatively correlated with innovation output, indicating that for every 1% increase in government subsidies, innovation investment will decrease by 1.643%. When the Sub_amount > 15.867, it promotes the firms' innovation output with a correlation coefficient of 3.154, indicating that for every 1% increase in government subsidies, innovation output increases by 3.154%. Hence, government subsidies of different intensities have different effects on the level of innovation inputs and outputs of this type of firm. Third, in the dynamic model, L.RDI and L.Patent promote firm innovation at the 5 and 1% levels, respectively, the effects of government subsidies on firms' innovation inputs and outputs follow the same trend as in the static model, but the threshold value and correlation significance are different from static ones. Therefore, the threshold effect of government subsidy on enterprise innovation input and innovation output will change with the influence of the dynamic situation. Li et al. (38) considered this as a kind of innovation inertia such that innovation performance in the past year will have an impact on innovation in the current year.

**Table 4:** Regression results of thresholds models.

**Variables**	**Static threshold**		**Dynamic threshold**	
	**RDI**	**Patent**	**RDI**	**Patent**
L.RDI			0.107^**^	
			(0.049)	
L.Patent				0.474^***^
				(0.033)
Below γ	−0.015	−1.643	−0.088^**^	−3.477^**^
	(0.031)	(1.863)	(0.043)	(1.510)
Above γ	0.497^*^	3.154	0.357^**^	24.835^***^
	(0.286)	(4.719)	(0.179)	(9.518)
Age	0.202^***^	2.520^***^	0.182^***^	0.567
	(0.018)	(0.456)	(0.018)	(0.481)
Size	−1.118^***^	3.448^*^	−0.896^***^	5.881^**^
	(0.094)	(1.786)	(0.100)	(2.369)
Lev	−0.044	10.872^**^	−0.059	7.703
	(0.169)	(4.603)	(0.184)	(5.850)
Roe	−0.060	8.101	0.034	12.170^*^
	(0.160)	(5.145)	(0.165)	(7.303)
Market	0.045	2.674^***^	0.058^*^	1.749^**^
	(0.031)	(0.653)	(0.035)	(0.888)
Holder	0.008^**^	−0.129	0.008^*^	−0.134
	(0.003)	(0.098)	(0.004)	(0.142)
Fix	0.005	0.001	0.010^***^	0.084
	(0.003)	(0.098)	(0.004)	(0.129)
State	−0.084	10.590^***^	−0.030	5.992^*^
	(0.084)	(1.878)	(0.062)	(3.294)
Threshold γ	17.591^***^	15.867^***^	16.897^***^	17.591^***^
	(0.465)	(1.224)	(0.436)	(0.352)
Observations	1,757	1,757	1,757	1,757

^***^, ^**^, and ^*^ denote *p* < 0.01, *p* < 0.05, and *p* < 0.1, respectively. Standard errors are in parentheses.

### 4.3. Heterogeneity analysis results

#### 4.3.1. Analysis of enterprise ownership level

From the results in Table 5 (for space reasons, the complete regression results of Tables 5–**8** including the control variables are provided in Appendix Tables A2–A4), the coefficients of the core explanatory variables do not change significantly with the inclusion of the control variables. Government subsidies significantly promote the innovation output of state biopharmaceutical firms at the 1% level, while there is a crowding-out effect on innovation inputs. For every 1% increase in government subsidy, innovation input decreases by 0.03%, while innovation output increases by 0.174%. For non-state enterprises, in all cases, government subsidies significantly promote innovation inputs and outputs of biopharmaceutical firms at the 1% level. For every 1% increase in government subsidy, innovation input increases by 0.168%, while innovation output increases by 0.359%. The impact of government subsidies on the innovation input and output of state and non-state biopharmaceutical companies is significantly different, and the incentive effect on non-state enterprises is more significant. According to the theory of corporate ownership, state enterprises serve as tools for government departments to achieve economic goals or policy objectives, and policy and subsidy preferences may not be fully used to increase R&D inputs; however, government subsidies have a significant incentive effect on firms' innovation output. Non-state enterprises are usually more active and innovative but lack financial investment. Therefore, government subsidies are beneficial in helping firms reduce R&D costs, which greatly promotes their innovation inputs and outputs. H3a is verified.

**Table 5:** Enterprise ownership sub–sample linear regression results.

**Variables**	**State enterprises**				**Non–state enterprises**			
	**(1)**	**(2)**	**(3)**	**(4)**	**(5)**	**(6)**	**(7)**	**(8)**
	**RDI**	**Patent**	**RDI**	**Patent**	**RDI**	**Patent**	**RDI**	**Patent**
Sub_amount	−0.040	0.194^***^	−0.030	0.174^***^	0.101^***^	0.401^***^	0.168^***^	0.359^***^
	(0.027)	(0.026)	(0.024)	(0.037)	(0.036)	(0.023)	(0.037)	(0.030)
Constant	2.598^***^	0.547	11.567^**^	−4.175^***^	0.895	−3.339^***^	15.722^***^	−7.568^***^
	(0.482)	(0.457)	(5.558)	(1.456)	(0.584)	(0.370)	(3.548)	(0.852)
Control var.	No	No	Yes	Yes	No	No	Yes	Yes
Observations	407	407	407	407	1,350	1,350	1,350	1,350

^***^, ^**^, and ^*^ denote *p* < 0.01, *p* < 0.05, and *p* < 0.1, respectively. Standard errors are in parentheses.

From the threshold regression results in Table 6, there is a significant threshold effect of government subsidies on the innovation inputs and outputs of both state and non-state biopharmaceutical firms, and hypothesis H3b is verified. Government subsidies significantly promote state enterprises' RDI when Sub_amount < 17.098, and inhibit RDI when Sub_amount > 17.098. The effects of government subsidies on state enterprises' innovation output show inconsistent trends in static and dynamic thresholds. However, in the dynamic threshold, L.Patent is negatively correlated with Patent at the 1% level, indicating that the inconsistency may be influenced by the lag effect of patent application. At this point, there is a suppression effect when Sub_amount < 15.555, and innovation output is significantly promoted when Sub_amount > 15.555. This indicates that when firms receive government subsidies above the threshold, there is some crowding-out effect on the innovation inputs of biopharmaceutical firms, but a significant incentive effect on the innovation output of state-owned biopharmaceutical firms. For non-state enterprises, government subsidies exhibit inhibitory effects on both innovation inputs and outputs when they are below the threshold and a significant incentive effect when they are above the threshold. This indicates that government subsidies provide great help to non-state enterprises with high R&D investment and fierce competition, which can significantly enhance enterprises' innovation vitality. Meanwhile, both L.RD and L.Patent have a significant positive effect in the current period, indicating that firms' innovation input and output have a significant continuous effect.

**Table 6:** Enterprise ownership sample threshold regression results.

**Variables**	**State enterprises**				**Non-state enterprises**			
	**Static threshold**		**Dynamic threshold**		**Static threshold**		**Dynamic threshold**	
	**RD**	**Patent**	**RDI**	**Patent**	**RDI**	**Patent**	**RDI**	**Patent**
L.RDI			−0.099^***^				0.106^**^	
			(0.019)				(0.044)	
L.Patent				0.460^***^				0.271^***^
				(0.010)				(0.033)
Below γ	0.041^***^	1.332^***^	0.021^**^	−5.109^***^	−0.009	−0.799	−0.140^***^	−7.274^***^
	(0.009)	(0.506)	(0.009)	(0.960)	(0.038)	(1.715)	(0.048)	(1.992)
Above γ	−0.099	0.534	−0.448^***^	6.098^**^	0.281	1.698	0.305	47.349^***^
	(0.087)	(2.492)	(0.130)	(2.876)	(0.271)	(3.245)	(0.193)	(9.484)
Control var.	Yes	Yes	Yes	Yes	Yes	Yes	Yes	Yes
Threshold γ	17.098^***^	16.706^***^	17.123^***^	15.555^***^	17.444^***^	16.095^***^	16.941^***^	17.444^***^
	(0.722)	(2.649)	(0.118)	(0.229)	(0.957)	(1.808)	(0.469)	(0.153)
Observations	364	364	364	364	1,281	1,281	1,281	1,281

^***^, ^**^, and ^*^ denote *p* < 0.01, *p* < 0.05, and *p* < 0.1, respectively. Standard errors are in parentheses.

#### 4.3.2. Analysis of regional differences

The full sample is divided into the more economically developed eastern regions and the less economically developed central and western regions. The former include Beijing, Tianjin, Hebei, Liaoning, Shanghai, Jiangsu, Zhejiang, Fujian, Shandong, Guangdong, and Hainan. The remaining regions are classified into the less economically developed central and western regions. From the results of Table 7, from the more economically developed eastern regions, government subsidies promote innovation input and output of enterprises at the 1% significance level, with correlation coefficients of 0.116 and 0.203, respectively. Indicating that for every 1% increase in government subsidies, innovation investment increases by 0.116%, while innovation output increases by 0.203%. From the less economically developed central and western regions, government subsidies promote the innovation input and output of biopharmaceutical enterprises at the 5 and 1% significance levels, respectively, with correlation coefficients of 0.112 and 0.323. Indicating that for every 1% increase in government subsidies, innovation investment increases by 0.112%, while innovation output increases by 0.323%. It can be found that government subsidies promote innovation inputs and outputs of biopharmaceutical companies in different regions to different degrees; H4a was validated.

**Table 7:** Regional sample linear regression results.

**Variables**	**Eastern regions**				**Central and western regions**			
	**(1)**	**(2)**	**(3)**	**(4)**	**(5)**	**(6)**	**(7)**	**(8)**
	**RDI**	**Patent**	**RDI**	**Patent**	**RDI**	**Patent**	**RDI**	**Patent**
Sub_amount	0.043	0.251^***^	0.116^***^	0.203^***^	0.093^*^	0.436^***^	0.112^**^	0.323^***^
	(0.032)	(0.018)	(0.034)	(0.027)	(0.054)	(0.031)	(0.049)	(0.040)
Constant	1.902^***^	−0.669^**^	17.495^***^	−6.425^***^	0.367	−4.058^***^	3.353	−4.943^***^
	(0.524)	(0.305)	(3.814)	(0.941)	(0.901)	(0.526)	(4.259)	(1.139)
Control var.	No	No	Yes	Yes	No	No	Yes	Yes
Observations	1,197	1,197	1,026	1,026	560	560	480	480

^***^, ^**^, and ^*^ denote *p* < 0.01, *p* < 0.05, and *p* < 0.1, respectively. Standard errors are in parentheses.

**Figure 2:**
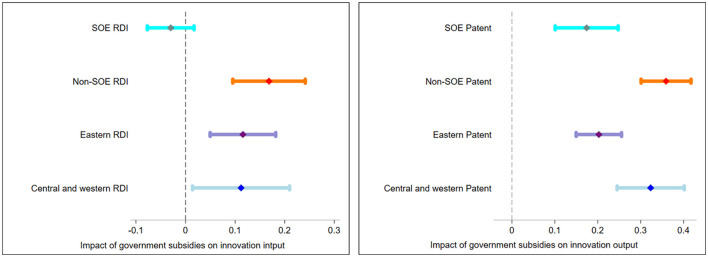
Impact of government subsidies on innovation inputs and outputs.

To understand the actual variability of the regional and ownership comparisons, we also visualize the different values obtained from the regressions in Figure 2. The center point indicates the impact of government subsidy, and the line segment indicates the confidence interval; hence, if the confidence interval intersects with the vertical line x = 0, it means that the coefficient is not significant. In the left panel, the influence coefficients of government subsidies on the innovation input of state enterprises, non-state enterprises, enterprises in the eastern and central and western regions are −0.03, 0.168, 0.116, and 0.112. The numerical changes are small, but can be clearly visualized through the images: the impact of government subsidies on innovation inputs differs for state and non-state biopharmaceutical firms, indicating a more significant promotion effect on non-state firms; at the same time, the differences between regions are indeed insignificant, and only the confidence intervals are larger in the central and western regions than in the eastern regions. In the right panel, the influence coefficients on their innovation output are 0.174, 0.359, 0.203, and 0.323. The confidence intervals do not overlap between state and non-state biopharmaceutical firms, suggesting that non-state firms are more strongly incentivized in terms of innovation output. The confidence interval for firms in the eastern regions slightly overlaps with those in the central and western regions, but a stronger effect is nonetheless visible for the latter regions.

According to Table 8, there is a significant threshold effect of government subsidies on innovation inputs and outputs of biopharmaceutical firms in both eastern and central and western regions, as verified by H4b. From the firms in the eastern regions, both L.RDI and L.Patent have significant effects on the current period at the 1% level, indicating that their effects on the regressions are not negligible. Thus, in the dynamic threshold, it is negatively correlated with RDI when Sub_amount < 17.465 and significantly positively correlated when Sub_amount > 17.465. It is positively correlated with Patent when Sub_amount < 15.46 and negatively correlated when Sub_amount > 15.46. Looking at the firms in the central and western regions, both L.RDI and L.Patent have significant effects on the current period at the 1% level, indicating that their effects on the regressions cannot be ignored. Therefore, in the dynamic threshold, it is significantly negatively correlated with RDI when Sub_amount < 15.944 and positively correlated when Sub_amount > 15.944. It is significantly negatively correlated with Patent when Sub_amount < 17.797 and significantly positively correlated when Sub_amount > 17.797. From the theoretical analysis of regional economy, the eastern regions are economically developed regions with strong resources, which can widely gather human and material resources and other capital to form enterprise clusters. Enterprises can rely on regional advantages to enhance their competitiveness, thus not relying exclusively on government subsidies to stimulate innovation inputs and outputs. The economically backward central and western regions are affected by the lack of regional resources and innovative talents, as well as the enterprises' own scale and profitability, which lead to weak innovation conditions and higher risk of innovation investment. Therefore, only when the government subsidies are sufficient to compensate for the lack of resources of enterprises in the regions are the enterprises more motivated to increase their innovation investment and output.

**Table 8:** Regional sample threshold regression results.

**Variables**	**Eastern regions**				**Central and western regions**			
	**Static threshold**		**Dynamic threshold**		**Static threshold**		**Dynamic threshold**	
	**RDI**	**Patent**	**RDI**	**Patent**	**RDI**	**Patent**	**RDI**	**Patent**
L.RDI			0.222^***^				0.162^***^	
			(0.059)				(0.016)	
L.Patent				0.477^***^				0.474^***^
				(0.027)				(0.006)
Below γ	−0.010	−1.699	−0.024	1.741	0.123^***^	−3.306^**^	−0.141^***^	−4.166^***^
	(0.028)	(1.191)	(0.028)	(2.441)	(0.036)	(1.669)	(0.045)	(0.949)
Above γ	0.698^**^	3.947	0.929^***^	−2.861	−0.260	−0.543	0.100	22.475^***^
	(0.300)	(2.643)	(0.285)	(6.029)	(0.225)	(3.192)	(0.108)	(3.561)
Control var.	Yes	Yes	Yes	Yes	Yes	Yes	Yes	Yes
Threshold γ	17.465^***^	16.183^***^	17.465^***^	15.460^***^	17.576^***^	15.613^***^	15.944^***^	17.797^***^
	(0.318)	(0.555)	(0.220)	(1.585)	(0.437)	(1.370)	(0.473)	(0.100)
Observations	1,197	1,197	1,197	1,197	560	560	560	560

^***^, ^**^, and ^*^ denote *p* < 0.01, *p* < 0.05, and *p* < 0.1, respectively. Standard errors are in parentheses.

## 5. Robustness tests

(i) Endogeneity. Endogeneity is an important issue in economic studies and can arise for various reasons. In addition to reducing potential bias due to endogeneity using a dynamic threshold model, we further refer to the heteroskedasticity-based instrumental variables proposed by Lewbel (78). For instance, Nie et al. (79) applied the method and allowed for IV estimation by exploring heteroscedasticity in the data without external instrumental variables. Consider *Y* = β_0_+β_1_*X*+β_2_*D*+μ and *X* = γ_0_+γ_1_*D*+ε, where Y is the outcome variable, X is the core explanatory variable, and D is the control variable. We can take a set of exogenous variables Z to construct an instrumental variable [*Z*−*E*(*Z*)]ε, as long as X and Z satisfy *E*(*Xμ*) = 0, *E*(*Xε*) = 0, *cov*(*Z*, μ, ε) = 0. Where, as mentioned by Lewbel (78), Z can be a subset or the full set of D. Here, we follow Nie et al. (79) and present the results associated with the full set of D; however, there is no qualitative change in the results when using a subset. According to Table 9, the instrumental variable results are consistent with those of previous studies.

**Table 9:** Robustness test results.

**Variables**	**Instrumental variable**		**Replace the RDI**		**Replace the patent**	
	**RDI**	**Patent**	**lnrd**	**lnrd**	**P1**	**P1**
Sub_amount	0.875^***^	0.395^***^	0.072^***^	0.029^**^	0.345^***^	0.280^***^
	(0.119)	(0.088)	(0.018)	(0.014)	(0.016)	(0.024)
Age	−0.018^**^	−0.031^***^		0.015		−0.011^*^
	(0.009)	(0.007)		(0.064)		(0.006)
Size	−0.859^***^	0.183^**^		0.449^***^		0.306^***^
	(0.120)	(0.084)		(0.051)		(0.045)
Lev	−0.711^**^	−0.075		−0.052		−0.3467^*^
	(0.295)	(0.196)		(0.119)		(0.203)
Roe	0.951^*^	0.531		0.055		−0.082
	(0.514)	(0.624)		(0.148)		(0.321)
Market	0.216^***^	−0.144^***^		0.056^**^		−0.026
	(0.042)	(0.034)		(0.022)		(0.025)
Holder	−0.013^***^	−0.010^***^		−0.003		0.002
	(0.004)	(0.004)		(0.003)		(0.002)
Fix	−0.009^**^	−0.010^***^		0.003		−0.010^***^
	(0.004)	(0.003)		(0.002)		(0.002)
State	0.214^*^	0.373^***^		−0.170^**^		0.437^***^
	(0.124)	(0.088)		(0.079)		(0.084)
Constant	7.419^***^	−6.090^***^	0.552^*^	−8.591^***^	−2.950^***^	−8.291^***^
	(1.254)	(1.001)	(0.297)	(1.302)	(0.271)	(0.796)
Observations	1,757	1,757	1,757	1,757	1,757	1,757

^***^, ^**^, and ^*^ denote *p* < 0.01, *p* < 0.05, and *p* < 0.1, respectively. Standard errors are in parentheses.

**Figure 3:**
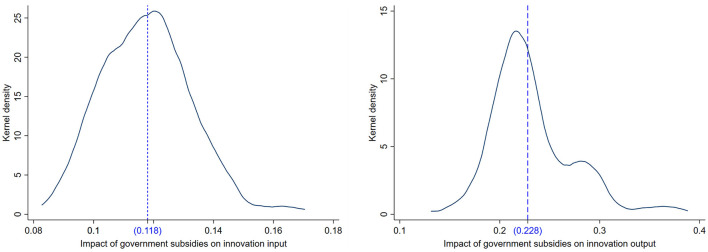
Randomly selected sub-samples' test results.

(ii) Alternative measures of explanatory variables. To ensure the reliability of the experimental results, we change the measures of innovation input and output. First, we replace *RDI* with total firm innovation input (in logarithmic form) (lnrd). Second, referring to Li et al. (38), who highlighted that invention patents are the most difficult to develop and most representative of innovation value, *Patent* is replaced with the number of invention patent applications (P1). The results in Table 9 indicate that the basic findings do not change significantly, again indicating that our estimation results are robust.

(iii) Randomly selected sub-samples. Although this study controls for firm heterogeneity characteristics as much as possible, there may still be unobservable factors affecting the experimental results. Therefore, the following random sub-sample test is constructed. A total of 500 experiments are randomly selected, and 80% of the entire sample is taken as a sub-sample each time. The fixed-effects and negative binomial model regressions were performed separately, and the coefficients of the key variables obtained from the regressions were presented in the kernel density. Figure 3 shows that the Sub_amount coefficients are all normally distributed, and our regression results of 0.118 (RDI) and 0.228 (Patent) are in the peak position, proving the generality and robustness of the basic conclusions.

## 6. Discussions and conclusions

Numerous studies have shown that, government subsidies play an important role in raising firms' level of innovation and enhancing their sustainability. To promote the development of the medical and pharmaceutical industry, the Chinese government has been increasing financial subsidies for innovation in biopharmaceutical firms. In recent years, influenced by the long-term goal of a “Healthy China” and the COVID-19 in the short term, China's biopharmaceutical industry has gradually improved the problem of “emphasis on generic, light on original” and “emphasis on quantity, light on quality”. The number of new drug development has been increasing, and the advantages in the field of traditional Chinese medicine have been gradually highlighted, increasing the international market share. Specifically, (1) government policies and subsidies continue to create the formation of a number of biopharmaceutical industry clusters, such as Henan Province to accelerate the medical Central Plains headquarters base, pharmaceutical intermediates and APIs “super factory”, rehabilitation aids industrial park and other key projects, building China Pharmaceutical Valley. Fujian Province actively invest special funds to cultivate innovation platforms for biopharmaceutical industry and build pharmaceutical innovation bases. (2) Biomedical technology is included in the high-tech fields supported by the key financial subsidy policy. Greater progress has been made in the development of new vaccines, bio-therapeutic technologies, rapid bio-detection, natural drug biosynthesis preparation and other fields. Meanwhile, the number of valid patents in the field of medical devices in China has increased year by year and has been accelerated in recent years. A total of 60 innovative medical device products were included in the special approval in 2021, and 28 products were successfully approved for marketing. (3) In 2021, the number of innovations in the field of biopharmaceuticals such as vaccines, antibody drugs, recombinant proteins, blood products, cell, and gene therapy is outstanding. The number of new listed products of recombinant protein drugs and blood products is six times higher than that in 2020. (4) The number of new Chinese medicines has climbed sharply, and the total number of Chinese medicines accepted by CDE (Center for Drug Evaluation) in 2021 was 1,360, of which 60 were new drug applications, an increase of 114.29%. Among them, Qinglung detoxification granules, Dampness defeating granules and Xuanlung defeating granules are all derived from ancient classical prescriptions, which are the fruitful transformation of effective prescriptions to fight against the COVID-19.

Currently, the cross-fertilization of biomedical technology with many industry sectors has driven new development trends in more areas. For example, (1) With the breakthrough of digital technology such as artificial intelligence, the importance of Internet and intelligent technology in the development process of biopharmaceutical industry is becoming more and more significant (80). On the one hand, a large number of global pharmaceutical companies have started to explore the combination of artificial intelligence and new drug development. At present, the main intelligent applications include conducting research on new drug design, physical and chemical property prediction, pharmaceutical analysis, disease diagnosis targets, drug combination use, etc. On the other hand, Internet healthcare, formed by the deep integration of the Internet and traditional medical services, is conducive to solving the contradiction between the imbalance of medical resources and people's increasing demand for health care. Especially, during the COVID-19, the epidemic-related services provided by Internet healthcare, such as online diagnosis and treatment and health information, effectively helped fight the epidemic.

(2) The idea of integrating biomedical and physical sciences is widely used to help explore new drugs and new therapeutic strategies (81, 82). Some researchers say that incorporating the physical properties of tumors and their surrounding tissues into existing biological and genetic models could increase understanding of cancer, thus leading cancer researchers down previously unknown paths, potentially leading to the discovery of new drugs and new treatment strategies.

Using panel data on biopharmaceutical firms in China, we draw the following conclusions. First, from an overall perspective, the linear model reveals a significant effect of government subsidies, and the incentive effect on innovation outputs is better than that on innovation inputs. There is a significant threshold effect of government subsidies on innovation inputs and outputs, suggesting an incentive interval in practice. This is consistent with the findings of Wei et al. (37), who specifically studied the relationship between government R&D subsidies and the innovation outputs of the medical equipment and instrumentation industry through the threshold model. The study focuses on examining the dynamic threshold model, which on the one hand solves part of the endogeneity problem, and on the other, it intuitively demonstrates that enterprise innovation activities are a continuous rather than intermittent process, which is similar to the innovation inertia found by new-energy firms (38). Compared with studies in other more mature high-tech industries (25, 83), China's biopharmaceutical industry is in the critical period, and the strength of firms and industrial agglomeration effect need to be further strengthened. With the recurring COVID-19 pandemic, the demand for key technologies and core equipment has been further highlighted. Government subsidies provide a significant incentive for biopharmaceutical companies to innovate.

Second, the incentive effect of government subsidies on firm innovation varies in terms of ownership. On the one hand, such subsidies significantly contribute to the innovation output of state enterprises as well as the innovation inputs and outputs of non-state enterprises. On the other hand, for state enterprises, when firms receive government subsidies above the threshold, there is some crowding-out effect on the innovation inputs of biopharmaceutical firms, but a significant incentive effect on innovation output. For non-state enterprises, when government subsidies reach an interval above the threshold, there is an incentive effect on both innovation inputs and outputs. Third, the incentive effect of government subsidies on firm innovation varies by regions. Government subsidies have a significant incentive effect in both the eastern and central and western regions of China. Firms in the latter regions seem to be more incentivized despite their natural locational disadvantages. From the threshold models, when government subsidies are in the above-threshold interval, they have more significant incentive effects on the innovation inputs of firms in the eastern regions, as well as on the innovation inputs and outputs of firms in the central and western regions; however, they fail to stimulate significant promotion effects when they are below the threshold interval.

## 7. Policy implications

The development of the biopharmaceutical industry has played an important role in improving the general health of population. It helps to meet the huge demand for medical services due to China's large population, aging population structure and longevity (84). Not only in treating chronic and serious diseases, but also in safeguarding human life and health in response to the COVID-19. With the advancement of medical technology, China's per capita life expectancy has been able to increase. At the same time, the integration of biomedical technologies with other fields has continuously improved the efficiency of traditional services. Thus, based on the conclusions, we draw the following policy implications from the perspectives of government and pharmaceutical firms.

From the government's perspective, the biopharmaceutical industry receives a much lower percentage of government subsidies than other high-tech industries (37). Thus, the implementation of policies and subsidies for biopharmaceutical firms should be enhanced to encourage R&D of new products and technologies of biopharmaceutical firms. Second, the government should consider the interval incentive effect of subsidies, change the principle of average distribution in the past, set a reasonable amount and intensity of subsidies for different firms, avoid excessive subsidies for pharmaceutical firms, and reduce the dependence of firms on government subsidies to a certain extent, so that finite subsidies can exert maximum influence. Third, the government should take differences in enterprise ownership in to account, optimize the allocation structure of innovation resources, give special subsidies to non-state biopharmaceutical firms, and help non-state biomedical enterprises reduce market costs. Furthermore, the guidance of government subsidies to the innovation investment of state biomedical enterprises should be strengthened, government subsidies allocated to state biopharmaceutical firms in stages, the utilization rate of the subsidies invested maximized, and the extrusion of funds from pharmaceutical firms minimized. The government should also consider the differences in regional innovation of biopharmaceutical firms, so that the subsidies can be combined with regional resources as much as possible to maximize its effect. Moreover, it should actively play the role of resource allocation, focus on the innovation of pharmaceutical firms in the central and western regions, and reasonably mobilize the consciousness of firms in these regions to develop innovation. Through demonstration and radiation, drive the development of pharmaceutical firms in the central and western regions, introduce leading firms to form a large-scale market, drive a large number of small and medium-sized firms in the pharmaceutical industry chain to settle in, achieve a good agglomeration effect, and enhance the innovation ability of firms in all aspects. Further, increase the publicity of the advantages of the aforementioned regions, help firms attract foreign investors to join, solve the problem of capital needs, and motivate pharmaceutical firms in these regions to innovate and enjoy innovation.

From the perspective of the biopharmaceutical firms, firstly, according to their unique requirements for innovation, firms should continuously enhance the capability of independent innovation and make full use of the preferential policies issued by government departments to develop continuous innovation. Firms can transform their current innovation patterns by setting up specialized research laboratories internally or by establishing innovation alliances between companies and research institutions. Second, biopharmaceutical companies should strengthen external publicity and convey more information about their internal development, such as profitability and innovation output level, in various ways, which will help to increase external understanding of the company and broaden the channels for obtaining government subsidies or external financing. Finally, biopharmaceutical companies should actively use the advantages of internal and external resources to enhance their conditions for innovation. State enterprises should make more efficient use of government subsidies and various preferential policies. Non-state enterprises should actively seek government subsidies and external financing to expand their resources for innovation. Firms in the eastern regions should make full use of the advantages of regional resources to increase their innovation capabilities. Firms in the central and western regions should grasp the development opportunities and actively use government subsidies to increase innovation inputs and expand outputs, so as to win market position and development future through innovation.

Our study has several limitations. First, all government subsidies received by firms were used in the process of data collection; however, the limited availability of data makes it difficult to obtain implicit government subsidies to firms, and research on specific types of government subsidies can be considered in future studies. Second, only the total number of patents was selected to measure the level of firms' innovation outputs, which may not be sufficiently comprehensive. Therefore, other measures can be added in future studies. Finally, owing to the limitations of the study methodology and models, it is difficult to explore the inconsistent sub-sample results in depth, and a detailed analysis will be conducted in the future to address regional differences.

## Data availability statement

The raw data supporting the conclusions of this article will be made available by the authors, without undue reservation.

## Author contributions

QLi and JD contributed to conception and design of the study. JD and QLiu collected the data. JD performed the statistical analysis and wrote the first draft of the manuscript. All authors contributed to manuscript revision, read, and approved the submitted version.
