# Supplementary material for: Impact of government subsidies on innovation of Chinese biopharmaceutical firms: Based on kink threshold model
Source: Front Public Health. 2023 Feb 23;11:1087830. doi: 10.3389/fpubh.2023.1087830 (PMC9995779; doi:10.3389/fpubh.2023.1087830)
Supplement: Supplementary file 1 [file Table_1.DOC]

# Appendix

**Table A1: Linear correlation test.**

|  | **RDI** | **Patent** | **Sub_a~t** | **Age** | **Size** | **Lev** | **Roe** | **Market** | **Holder** | **Fix** | **State** |
| --- | --- | --- | --- | --- | --- | --- | --- | --- | --- | --- | --- |
| **RDI** | 1.000 |  |  |  |  |  |  |  |  |  |  |
| **Patent** | 0.287*** | 1.000 |  |  |  |  |  |  |  |  |  |
| **Sub_amount** | 0.198*** | 0.320*** | 1.000 |  |  |  |  |  |  |  |  |
| **Age** | -0.111*** | 0.090*** | 0.270*** | 1.000 |  |  |  |  |  |  |  |
| **Size** | -0.103*** | 0.294*** | 0.617*** | 0.508*** | 1.000 |  |  |  |  |  |  |
| **Lev** | -0.166*** | 0.133*** | 0.244*** | 0.311*** | 0.419*** | 1.000 |  |  |  |  |  |
| **Roe** | 0.089*** | 0.024 | 0.127*** | 0.046* | 0.131*** | -0.153*** | 1.000 |  |  |  |  |
| **Market** | 0.223*** | -0.082*** | 0.058** | -0.038 | -0.059** | -0.364*** | 0.233*** | 1.000 |  |  |  |
| **Holder** | -0.097*** | -0.020 | -0.034 | -0.075*** | 0.038 | -0.043* | 0.218*** | 0.071*** | 1.000 |  |  |
| **Fix** | -0.042* | -0.118*** | -0.092*** | -0.017 | -0.201*** | 0.083*** | -0.098*** | -0.082*** | -0.001 | 1.000 |  |
| **State** | -0.086*** | 0.116*** | 0.094*** | 0.538*** | 0.253*** | 0.217*** | 0.012 | -0.172*** | 0.090*** | 0.002 | 1.000 |
|  |  | **Mean** | **Sub_a~t** | **Age** | **Size** | **Lev** | **Roe** | **Market** | **Holder** | **Fix** | **State** |
| **VIF** |  | 1.510 | 1.670 | 1.890 | 2.400 | 1.510 | 1.160 | 1.260 | 1.110 | 1.090 | 1.500 |
| **1/VIF** |  |  | 0.599 | 0.529 | 0.416 | 0.664 | 0.862 | 0.792 | 0.904 | 0.913 | 0.665 |
| *Note: *** p<0.01, ** p<0.05, * p<0.1* | | | | | | | | | | | |

**Table A2: Enterprise ownership sub-sample linear regression results.**

| **Variables** |  | **State enterprises** | | | |  | **Non-state enterprises** | | | |
| --- | --- | --- | --- | --- | --- | --- | --- | --- | --- | --- |
|  |  | **(1)** | **(2)** | **(3)** | **(4)** |  | **(5)** | **(6)** | **(7)** | **(8)** |
|  |  | **RDI** | **Patent** | **RDI** | **Patent** |  | **RDI** | **Patent** | **RDI** | **Patent** |
| **Sub_amount** |  | -0.040 | 0.200*** | -0.030 | 0.180*** |  | 0.101*** | 0.401*** | 0.169*** | 0.359*** |
|  |  | (0.027) | (0.026) | (0.024) | (0.037) |  | (0.036) | (0.023) | (0.037) | (0.030) |
| **Age** |  |  |  | 0.032 | -0.004 |  |  |  | 0.197 | -0.051*** |
|  |  |  |  | (0.265) | (0.012) |  |  |  | (0.130) | (0.006) |
| **Size** |  |  |  | -0.464** | 0.244*** |  |  |  | -0.837*** | 0.243*** |
|  |  |  |  | (0.180) | (0.077) |  |  |  | (0.158) | (0.049) |
| **Lev** |  |  |  | -1.145** | 0.646 |  |  |  | 0.248 | 0.156 |
|  |  |  |  | (0.516) | (0.412) |  |  |  | (0.345) | (0.219) |
| **Roe** |  |  |  | 1.471** | 1.693** |  |  |  | -0.028 | 0.427 |
|  |  |  |  | (0.605) | (0.733) |  |  |  | (0.414) | (0.331) |
| **Market** |  |  |  | 0.046 | -0.285*** |  |  |  | 0.177** | -0.105*** |
|  |  |  |  | (0.162) | (0.082) |  |  |  | (0.077) | (0.025) |
| **Holder** |  |  |  | 0.014 | 0.008** |  |  |  | 0.011* | -0.001 |
|  |  |  |  | (0.009) | (0.004) |  |  |  | (0.006) | (0.002) |
| **Fix** |  |  |  | 0.027*** | -0.039*** |  |  |  | 0.001 | 0.006** |
|  |  |  |  | (0.007) | (0.005) |  |  |  | (0.005) | (0.003) |
| **Constant** |  | 2.598*** | 0.547 | 11.567** | -4.178*** |  | 0.896 | -3.339*** | 15.722*** | -7.568*** |
|  |  | (0.482) | (0.457) | (5.558) | (1.456) |  | (0.584) | (0.370) | (3.548) | (0.852) |
| **Observations** |  | 407 | 407 | 407 | 407 |  | 1,350 | 1,350 | 1,350 | 1,350 |

Note: ***, **, and * denote p<0.01, p<0.05 and p<0.1, respectively. Standard errors are in parentheses.

**Table A3: Enterprise ownership sample threshold regression results.**

| **Variables** |  | **State enterprises** | | | |  | **Non-state enterprises** | | | |
| --- | --- | --- | --- | --- | --- | --- | --- | --- | --- | --- |
|  |  | **Static threshold** | | **Dynamic threshold** | |  | **Static threshold** | | **Dynamic threshold** | |
|  |  | **RD** | **Patent** | **RDI** | **Patent** |  | **RDI** | **Patent** | **RDI** | **Patent** |
| **L.RDI** |  |  |  | -0.099*** |  |  |  |  | 0.106** |  |
|  |  |  |  | (0.019) |  |  |  |  | (0.044) |  |
| **L.Patent** |  |  |  |  | 0.460*** |  |  |  |  | 0.271*** |
|  |  |  |  |  | (0.010) |  |  |  |  | (0.033) |
| **Below γ** |  | 0.041*** | 1.332*** | 0.021** | -5.109*** |  | -0.009 | -0.799 | -0.140*** | -7.274*** |
|  |  | (0.009) | (0.506) | (0.009) | (0.960) |  | (0.038) | (1.715) | (0.048) | (1.992) |
| **Above γ** |  | -0.099 | 0.534 | -0.448*** | 6.098** |  | 0.281 | 1.698 | 0.305 | 47.349*** |
|  |  | (0.087) | (2.492) | (0.130) | (2.876) |  | (0.271) | (3.245) | (0.193) | (9.484) |
| **Age** |  | 0.041*** | 1.918*** | -0.010 | 1.455*** |  | 0.221*** | 2.213*** | 0.204*** | 0.664 |
|  |  | (0.007) | (0.354) | (0.010) | (0.337) |  | (0.021) | (0.490) | (0.021) | (0.562) |
| **Size** |  | 0.030 | 3.038* | 0.253*** | 7.098*** |  | -1.132*** | 5.978*** | -0.793*** | 9.106*** |
|  |  | (0.063) | (1.829) | (0.089) | (1.727) |  | (0.101) | (1.787) | (0.107) | (2.795) |
| **Lev** |  | 0.617*** | 15.147* | -1.028*** | 6.815 |  | -0.181 | -2.802 | -0.373** | 2.755 |
|  |  | (0.113) | (8.415) | (0.277) | (11.879) |  | (0.178) | (4.428) | (0.188) | (5.715) |
| **Roe** |  | 1.207*** | 10.577** | 1.791*** | -20.610*** |  | -0.220 | 6.672 | -0.289 | 6.452 |
|  |  | (0.105) | (4.931) | (0.219) | (5.545) |  | (0.178) | (5.305) | (0.189) | (7.862) |
| **Market** |  | 0.042 | 8.692*** | 0.297*** | 1.864 |  | 0.033 | 1.278 | 0.071* | 1.932* |
|  |  | (0.055) | (1.336) | (0.047) | (1.783) |  | (0.033) | (0.821) | (0.038) | (1.094) |
| **Holder** |  | 0.019*** | 0.378*** | 0.001 | 1.051*** |  | 0.009** | -0.071 | 0.007 | -0.237 |
|  |  | (0.002) | (0.041) | (0.003) | (0.085) |  | (0.004) | (0.107) | (0.005) | (0.167) |
| **Fix** |  | 0.007*** | 0.202*** | 0.016*** | 0.622*** |  | 0.003 | 0.208** | 0.009** | 0.440*** |
|  |  | (0.002) | (0.044) | (0.003) | (0.087) |  | (0.004) | (0.102) | (0.004) | (0.146) |
| **Threshold γ** |  | 17.098*** | 16.706*** | 17.123*** | 15.555*** |  | 17.444*** | 16.095*** | 16.941*** | 17.444*** |
|  |  | (0.722) | (2.649) | (0.118) | (0.229) |  | (0.957) | (1.808) | (0.469) | (0.153) |
| **Observations** |  | 364 | 364 | 364 | 364 |  | 1281 | 1281 | 1281 | 1281 |

Note: ***, **, and * denote p<0.01, p<0.05 and p<0.1, respectively. Standard errors are in parentheses.

**Table A4: Regional sample linear regression results.**

| **Variables** | | **Eastern regions** | | | | |  | **Central and western regions** | | | | |
| --- | --- | --- | --- | --- | --- | --- | --- | --- | --- | --- | --- | --- |
|  |  | **(1)** | **(2)** | **(3)** | **(4)** | |  | **(5)** | | **(6)** | **(7)** | **(8)** |
|  |  | **RDI** | **Patent** | **RDI** | | **Patent** |  | **RDI** | **Patent** | | **RDI** | **Patent** |
| **Sub_amount** |  | 0.043 | 0.251*** | 0.116*** | | 0.203*** |  | 0.093* | 0.437*** | | 0.112** | 0.323*** |
|  |  | (0.032) | (0.018) | (0.034) | | (0.027) |  | (0.054) | (0.031) | | (0.049) | (0.040) |
| **Age** |  |  |  | 0.030 | | -0.021*** |  |  |  | | 0.797*** | -0.060*** |
|  |  |  |  | (0.115) | | (0.007) |  |  |  | | (0.160) | (0.009) |
| **Size** |  |  |  | -0.838*** | | 0.279*** |  |  |  | | -0.454*** | 0.193*** |
|  |  |  |  | (0.168) | | (0.052) |  |  |  | | (0.163) | (0.066) |
| **Lev** |  |  |  | -0.243 | | 0.383 |  |  |  | | 0.298 | 0.071 |
|  |  |  |  | (0.368) | | (0.247) |  |  |  | | (0.553) | (0.305) |
| **Roe** |  |  |  | 0.287 | | 1.120*** |  |  |  | | -0.800 | 0.797* |
|  |  |  |  | (0.545) | | (0.400) |  |  |  | | (0.541) | (0.469) |
| **Market** |  |  |  | 0.346*** | | -0.081*** |  |  |  | | 0.088 | -0.180*** |
|  |  |  |  | (0.113) | | (0.031) |  |  |  | | (0.101) | (0.038) |
| **Holder** |  |  |  | 0.007 | | 0.013*** |  |  |  | | 0.001 | -0.011*** |
|  |  |  |  | (0.007) | | (0.002) |  |  |  | | (0.009) | (0.003) |
| **Fix** |  |  |  | 0.010* | | -0.003 |  |  |  | | 0.004 | -0.015*** |
|  |  |  |  | (0.005) | | (0.003) |  |  |  | | (0.007) | (0.004) |
| **State** |  |  |  | -0.052 | | 0.357*** |  |  |  | | -1.185** | 0.108 |
|  |  |  |  | (0.203) | | (0.097) |  |  |  | | (0.561) | (0.139) |
| **Constant** |  | 1.902*** | -0.669** | 17.495*** | | -6.425*** |  | 0.367 | -4.058*** | | 3.353 | -4.943*** |
|  |  | (0.524) | (0.305) | (3.814) | | (0.941) |  | (0.901) | (0.526) | | (4.259) | (1.139) |
| **Observations** |  | 1,197 | 1,197 | 1,026 | | 1,026 |  | 560 | 560 | | 480 | 480 |

Note: ***, **, and * denote p<0.01, p<0.05 and p<0.1, respectively. Standard errors are in parentheses.

**Table A5: Regional sample threshold regression results.**

|  |  | **Eastern regions** | | | |  | **Central and western regions** | | | |
| --- | --- | --- | --- | --- | --- | --- | --- | --- | --- | --- |
| **Variables** |  | **Static threshold** | | **Dynamic threshold** | |  | **Static threshold** | | **Dynamic threshold** | |
|  |  | **RDI** | **Patent** | **RDI** | **Patent** |  | **RDI** | **Patent** | **RDI** | **Patent** |
| **L.RDI** |  |  |  | 0.222*** |  |  |  |  | 0.162*** |  |
|  |  |  |  | (0.059) |  |  |  |  | (0.016) |  |
| **L.Patent** |  |  |  |  | 0.477*** |  |  |  |  | 0.474*** |
|  |  |  |  |  | (0.027) |  |  |  |  | (0.006) |
| **Below γ** |  | -0.010 | -1.699 | -0.024 | 1.741 |  | 0.123*** | -3.306** | -0.141*** | -4.166*** |
|  |  | (0.028) | (1.191) | (0.028) | (2.441) |  | (0.036) | (1.669) | (0.045) | (0.949) |
| **Above γ** |  | 0.698** | 3.947 | 0.929*** | -2.861 |  | -0.260 | -0.543 | 0.100 | 22.475*** |
|  |  | (0.300) | (2.643) | (0.285) | (6.029) |  | (0.225) | (3.192) | (0.108) | (3.561) |
| **Age** |  | 0.232*** | 2.615*** | 0.192*** | 0.665 |  | 0.167*** | 2.013*** | 0.041*** | 0.629* |
|  |  | (0.021) | (0.500) | (0.021) | (0.465) |  | (0.018) | (0.461) | (0.013) | (0.361) |
| **Size** |  | -1.310*** | 2.725 | -1.118*** | 4.152 |  | -1.040*** | 1.500** | -0.299*** | 9.396*** |
|  |  | (0.100) | (2.045) | (0.125) | (2.569) |  | (0.097) | (0.760) | (0.068) | (1.705) |
| **Lev** |  | -0.104 | 15.678*** | -0.328 | 6.896 |  | -0.224 | 10.500*** | 0.430** | -8.966 |
|  |  | (0.183) | (5.116) | (0.215) | (6.896) |  | (0.223) | (3.572) | (0.199) | (5.717) |
| **Roe** |  | 0.301 | 3.713 | 0.217 | 16.042** |  | -0.373*** | 2.592 | -0.175* | -12.338** |
|  |  | (0.216) | (6.017) | (0.219) | (7.819) |  | (0.126) | (4.710) | (0.093) | (5.789) |
| **Market** |  | 0.098** | 3.860*** | 0.158*** | 0.913 |  | 0.072*** | 2.895*** | 0.142*** | 0.090 |
|  |  | (0.038) | (1.112) | (0.040) | (1.179) |  | (0.024) | (0.521) | (0.027) | (0.665) |
| **Holder** |  | 0.006** | -0.261*** | 0.003 | -0.538*** |  | -0.016*** | -0.206*** | 0.005* | -0.030 |
|  |  | (0.003) | (0.095) | (0.005) | (0.142) |  | (0.004) | (0.057) | (0.003) | (0.095) |
| **Fix** |  | 0.006* | 0.006 | 0.014*** | 0.149 |  | 0.001 | -0.117 | 0.016*** | 0.266** |
|  |  | (0.004) | (0.091) | (0.004) | (0.135) |  | (0.003) | (0.095) | (0.004) | (0.119) |
| **State** |  | -0.028 | 8.877*** | 0.031 | -4.216*** |  | -0.316* | 2.390** | 0.044 | 15.369*** |
|  |  | (0.060) | (2.347) | (0.032) | (0.766) |  | (0.183) | (1.054) | (0.075) | (2.623) |
| **Threshold γ** |  | 17.465*** | 16.183*** | 17.465*** | 15.460*** |  | 17.576*** | 15.613*** | 15.944*** | 17.797*** |
|  |  | (0.318) | (0.555) | (0.220) | (1.585) |  | (0.437) | (1.370) | (0.473) | (0.100) |
| **Observations** |  | 1197 | 1197 | 1197 | 1197 |  | 560 | 560 | 560 | 560 |

Note: ***, **, and * denote p<0.01, p<0.05 and p<0.1, respectively. Standard errors are in parentheses.
